# 1,2-Dilinoleoyl-*sn*-glycero-3-phosphoethanolamine ameliorates age-related spatial memory deterioration by preventing neuronal cell death

**DOI:** 10.1186/1744-9081-6-52

**Published:** 2010-09-13

**Authors:** Takahiro Yaguchi, Tetsu Nagata, Tomoyuki Nishizaki

**Affiliations:** 1 Division of Bioinformation, Department of Physiology, Hyogo College of Medicine, 1-1 Mukogawa-cho, Nishinomiya 663-8501, Japan

## Abstract

**Background:**

Accumulating evidence has pointed that a variety of lipids could exert their beneficial actions against dementia including Alzheimer disease and age-related cognitive decline via diverse signaling pathways. Endoplasmic reticulum (ER) stress-induced neuronal apoptosis, on the other hand, is a critical factor for pathogenesis of neurodegenerative diseases such as Alzheimer disease and Parkinson disease, senile dementia, and ischemic neuronal damage. The present study examined the effects of 1,2-dilinoleoyl-*sn*-glycero-3-phosphoethanolamine (DLPhtEtn), a phospholipid, on ER stress-induced neuronal death and age-related cognitive disorders.

**Methods:**

PC-12 cell viability was assayed before and after treatment with amyloid-β_1-40 _peptide or thapsigargin in the presence and absence of DLPhtEtn. A series of behavioral tests were performed for senescence-accelerated mouse-prone 8 (SAMP8) mice after 7-month oral administration with polyethylene glycol (PEG) or DLPhtEtn and then, the number of hippocampal neurons was counted.

**Results:**

Amyloid-β_1-40 _peptide or thapsigargin is capable of causing ER stress-induced apoptosis. DLPhtEtn (30 μM) significantly inhibited PC-12 cell death induced by amyloid-β_1-40 _peptide or thapsigargin. In the water maze test, oral administration with DLPhtEtn (1 mg/kg) for 7 months (three times a week) significantly shortened the prolonged retention latency for SAMP8 mice. In contrast, DLPhtEtn had no effect on the acquisition and retention latencies in both the open field test and the passive avoidance test for SAMP8 mice. Oral administration with DLPhtEtn (1 mg/kg) for 7 months prevented a decrease in the number of hippocampal neurons for SAMP8 mice.

**Conclusion:**

The results of the present study show that DLPhtEtn ameliorates age-related spatial memory decline without affecting motor activities or fear memory, possibly by protecting hippocampal neuronal death. DLPhtEtn, thus, might exert its beneficial action against senile dementia and neurodegenerative diseases such as Alzheimer disease.

## Background

Endoplasmic reticulum (ER) stress, i.e., intraluminal accumulation of unfolded proteins, induces apoptosis by activating caspase-12 for mice and rats/caspase-4 for humans, and in turn, the effector caspase-3 [1-3]. Lines of evidence have pointed to ER stress-induced neuronal apoptosis as a critical factor for pathogenesis of neurodegenerative diseases such as Alzheimer disease and Parkinson disease, senile dementia, and ischemic neuronal damage [4-9].

Interestingly, gene expression alterations in the sphingolipid metabolism pathways such as upregulation of ceramide and downregulation of glycosphingolipids and sphingosine 1-phosphate (S1P), are identified during progression of dementia and Alzheimer's disease [10]. Increased ceramide and reduced S1P are found with the Alzheimer brains [11]. Soluble oligomers of amyloid-β peptide activates cytosolic calcium-dependent phospholipase A_2 _and sphingomyelinase, causing a ceramide rise to induce neuronal death responsible for Alzheimer disease [12]. Sphingolipids, thus, may be a mediator for progression of Alzheimer disease [13-15].

In contrast, the phospholipid phosphatidylcholine and its metabolites such as *cis*-unsaturated free fatty acids and lysophospholipids might enhance cognitive functions. 1,2-Dilinoleoyl-*sn*-glycero-3-phosphocholine (DLPhtCho) or 1-palmitoyl-2-oleoyl-*sn*-glycero-3-phosphocholine improves scopolamine-induced impairment of spatial learning and memory for rats or mild cognitive impairment/dementia for humans [16,17]. Rat hippocampal synaptic transmission is facilitated via a pathway linked to phospholipase A_2_, that hydrolyzes phosphatidylcholine into *cis*-unsaturated free fatty acids and lysophosphatidylcholine [18]. The *cis*-unsaturated free fatty acids, arachidonic, linoleic, linolenic, and oleic acid, could facilitate hippocampal synaptic transmission, like long-term potentiation (LTP), a cellular model of learning and memory, by enhancing activity of nicotinic acetylcholine (ACh) receptors or α-amino-3-hydroxy-5-methyl-4-isoxazolepropionic acid receptors via a protein kinase C (PKC) pathway or a Ca^2+^/calmodulin-dependent protein kinase II pathway [19-25]. Arachidonic acid serves as a retrograde messenger of LTP [26]. Lysophosphatidylcholine and lysophosphatidic acid could also facilitate hippocampal synaptic transmission by targeting nicotinic ACh receptors [27,28]. Moreover, the linoleic acid derivative 8-[2-(2-pentyl-cyclopropylmethyl)-cyclopropyl]-octanoic acid (DCP-LA) directly and selectively activates PKC-ε, thereby enhancing activity of presynaptic α7 ACh receptors involving glutamate release, and then leading to facilitation of hippocampal synaptic transmission [29-31]. DCP-LA ameliorates memory deficits in rat models treated with amyloid-β peptide or scopolamine [32]. DCP-LA neutralizes mutant amyloid β peptide-induced impairment of LTP and spatial learning [33] or protects neurons from oxidative stress-induced apoptosis by inhibiting caspase-3/-9 activation [34]. DCP-LA, alternatively, improves age-related spatial learning deterioration in SAMP8 mice [35]. A variety of lipids, thus, could exert their beneficial actions against dementia including Alzheimer disease and age-related cognitive decline via diverse signaling pathways. The effect of the phospholipid DLPhtEtn on cognitive disorders, however, is far from understanding.

SAMP mice such as SAMP1, SAMP2, SAMP3, SAMP6, SAMP7, SAMP8, and SAMP9 are widely used as a murine model of accelerated senescence, and senescence-accelerated mouse-resistant (SAMR) mice such as SAMR1 and SAMR2, that reveal normal aging, are used as a control for SAMP mice [36,37]. SAMP mice are shown to exhibit learning and memory disorders along age-related reduction of choline acetyltransferase activity [38-40]. The present study aimed at understanding the effect of DLPhtEtn on neuronal death and age-related impairment of spatial learning and memory using PC-12 cells and SAMP8 mice.

We show here that DLPhtEtn ameliorates age-related spatial memory deterioration, at least in part by inhibiting hippocampal neuronal death.

## Methods

### Animal care

All procedures have been approved by the Animal Care and Use Committee at Hyogo College of Medicine and were in compliance with the National Institutes of Health Guide for the Care and Use of Laboratory Animals.

### Cell culture

PC-12 cells, obtained from the RIKEN Cell Bank (Tsukuba, Japan), were cultured in Dulbecco's modified Eagle's medium containing 10% (v/v) fetal bovine serum and 10% (v/v) horse serum, penicillin (final concentration, 100 U/ml), and streptomycin (final concentration, 0.1 mg/ml) in a humidified atmosphere of 5% CO_2 _and 95% air at 37°C.

### Assay of cell viability

PC-12 cells were treated with amyloid-β_1-40 _peptide (Peptide Institute Inc., Osaka, Japan) or thapsigargin (Wako, Osaka, Japan) in the presence and absence of phospholipids such as DLPhtEtn (Avanti Polar Lipid, Inc., Alabaster, AL, USA), 1-linoleoyl-2-palmitoyl-*sn*-glycero-3-phosphoethanolamine (LPPhtEtn) (Avanti Polar Lipid, Inc.), 1,2-dioleoyl-*sn*-glycero-3-phosphoethanolamine (DOPhtEtn) (Avanti Polar Lipid, Inc.), 1,2-dipalmitoyl-*sn*-glycero-3-phosphoethanolamine (DPPhtEtn) (Avanti Polar Lipid, Inc.), 1,2-diheptadecanoyl-*sn*-glycero-3-phosphoethanolamine (DHPhtEtn) (Avanti Polar Lipid, Inc.), 1,2-distearoyl -*sn*-glycero-3-phosphoethanolamine (DSPhtEtn) (Avanti Polar Lipid, Inc.), or DLPhtCho (Avanti Polar Lipid, Inc.), dissolved with PEG, and cell viability was evaluated by a dye staining method using 3-(4,5-dimethyl-2-thiazolyl)-2,5-diphenyl-2H-tetrazolium bromide (MTT) (DOJINDO, Kumamoto, Japan) by the method as previously described [41].

### Animal preparation

Male SAMP8 and SAMR1 mice (age, 4 weeks) were obtained from Takeda Pharmaceutical Co. (Osaka, Japan), and 17 SAMP8 mice and 5 SAMR1 mice were used for experiments. Mice were individually housed in cages at 23 ± 1°C, with a 12-h light/dark cycle (lighting up at 7:00 a.m.), and had free access to pellet food and water. DLPhtEtn dissolved with PEG (final volume, 0.1 ml) or PEG alone (final volume, 0.1 ml) was orally administered (per os) to mice three times a week (Monday, Wednesday, and Friday) for 7 months prior to experiments. All the tests were carried out between 9:00 a.m. and 5:00 p.m.. Behavioral test batteries were run in the order of open field test, water maze test, and passive avoidance test using a single mouse, from least invasive to more invasive, to minimize the effects of the training history, as previously described [35].

### Open field test

Open field box, that is a cube constructed of 30 cm × 30 cm plastic plate, covered with wooden box to shut out light, was prepared for an open field test. The open-field arena was lighted up at 110 lx by an incandescent lamp fixed on the roof of the box. To avoid outer noise, a fan, producing a noise of 45 dB, was fixed on the wall of the box. Horizontal infrared beams run 2 cm above the floor with a 10-cm interval from two bidirectional walls, making nine cross stripes, and 4.5 cm above the floor with a 2.5-cm interval from one wall. The locomotion and rearing activity was assessed by counting the number for mice to cross the beams at 2 and 4.5 cm, respectively. Mice were allowed to freely move in the open-field arena. The initial half time of the test was performed under the light conditions, and the latter half time of the test under the dark conditions.

### Water maze test

A circular plastic water tank with 90 cm in diameter and 36 cm in deep was used for a water maze test. The entire inside of the pool was painted black, and the pool was filled up to 20 cm from the bottom with water darkened by India ink at 22°C. A platform (11 cm in diameter) painted black was placed into water, the top sinking 0.5 cm below water surface. The pool was put in a test room, where there were spatial cues that mice were able to see from the pool. The position of the marks remained unchanged throughout testing. A platform was located in the constant position, i.e., in the middle of one quadrant, equidistant from the center and edge of the pool. Mice were placed into the water, facing the wall of the pool at one of 5 positions selected at random, and time from start to escape onto the platform was measured. When succeeded, mice were allowed to stay on the platform for 10 s. When mice failed to find the platform within 90 s, the trial was stopped and mice were put on the platform for 10 s. Two trials were carried out a day, and the second trial began 2 min after the end of the first trial. Mice received the task for consecutive 8 days, and the mean acquisition latency (time form the start to arrival onto the plate) from consecutive 2 days was calculated. Seven days later, the platform was removed and the retention latency (time from the start to arrival to the place where the platform had been set) was measured.

### Passive avoidance test

A two-compartment step-through passive avoidance apparatus; a front illuminated chamber (10 cm × 10 cm floor and wall with 20 cm in height) and a rear dark chamber (10 cm × 10 cm floor and wall with 20 cm in height) was used for a passive avoidance test. Each chamber was separated by a guillotine door and grids were attached on both the floors. On the first day mice were put in the light chamber, and the guillotine door was locked when mice entered the dark chamber followed by an electrical stimulation of 0.36 mA for 3 s to feet, thereafter leaving mice in the chamber for 30 s. Then, mice were transferred in the light chamber again, and staying time in the light chamber was measured as acquisition latency. Next day (24 h later) mice were put in the light chamber, and staying time in the light chamber was measured as retention latency.

### Measurement of body weight and brain weight

The body weight was measured in SAMR1 and SAMP8 mice every month after oral administration with PEG or DLPhtEtn (1 mg/kg). Mice, that had taken a series of behavioral tests, were sacrificed and removed the brains, and then, each brain weight was measured.

### Measurement of hippocampal area

The brain, removed after behavioral tests, was fixed with formaldehyde and frozen in powdered dry ice. Then, coronal sections of the brain was made at a thickness of 18 μm using a cryostat (LEICA CM 1800, Germany), and a series of 8 sections from the beginning of the section on the rostral side, where the hippocampus was detectable, towards the caudal side were used for analysis. Hippocampal area both on the left and right sides was measured using a computer-based image analysis system (Image J, NIH, Bethesda, MD, USA), and areas from 8 sections per a mouse were summated.

### Counting of hippocampal neurons

All of 8 coronal sections prepared were reacted with a mouse monoclonal antibody against NeuN (1:500) (CHEMICON, Billerica, MA, USA), a marker for neurons, followed by an Alexa Fluor 488-conjugated secondary antibody (1:500) (Invitogen, Carlsbad, CA, USA). The number of neurons reactive to an anti-NeuN antibody was counted in the consistent area (142 μm × 192 μm) of the hippocampal CA1 region on both the left and right side from 8 sections per a mouse and summated.

### Statistical analysis

Statistical analysis was carried out using analysis of variance (ANOVA) followed by Fisher's Protected Least Significant Difference (PLSD) test as a post-hoc test and unpaired *t*-test.

## Results

### DLPhtEtn prevents PC-12 cell death induced by amyloid-β_1-40 _peptide or thapsigargin

**Figure 1:**
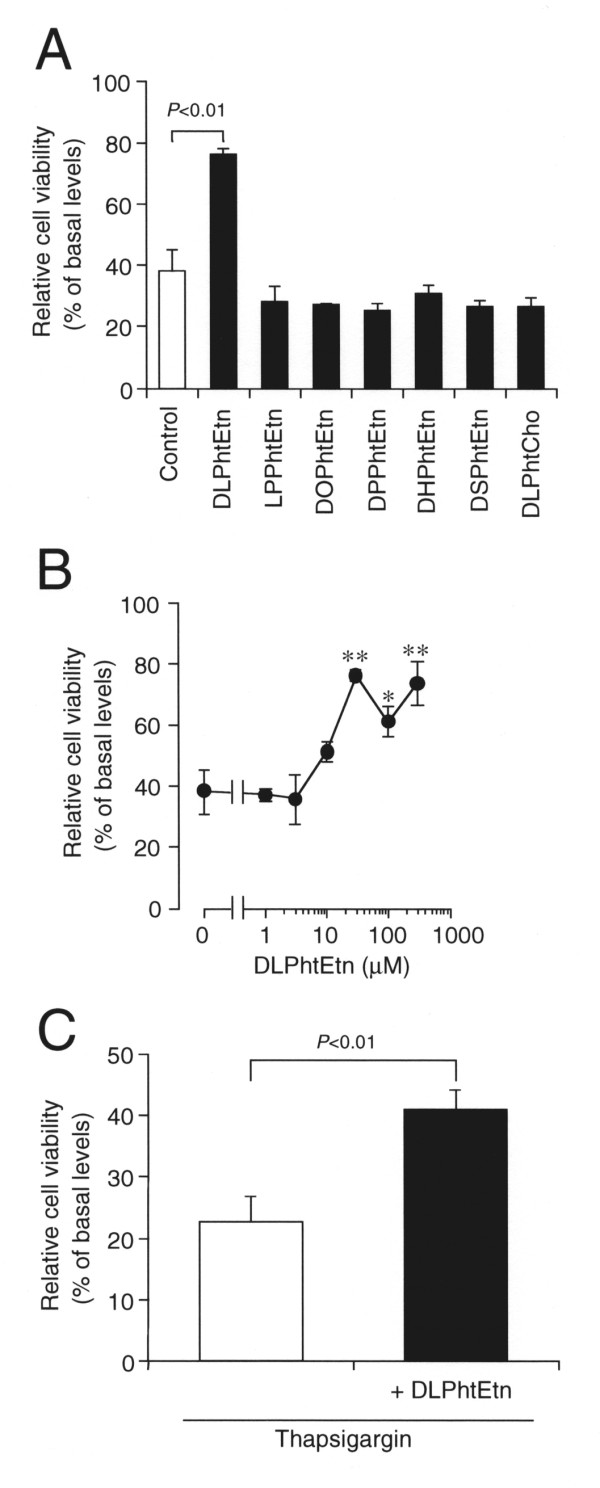
**The effect of DLPhtEtn on PC-12 cell death induced by amyloid-β_1-40 _peptide or thapsigargin**. (**A**) PC-12 cells were treated with amyloid-β_1-40 _peptide (5 μM) in the absence (Control) and presence of phospholipids (30 μM) as indicated in serum-free extracellular solution for 48 h, and cell viability was assayed. Data represents the mean (± SEM) percentage of basal levels (MTT intensities of cells untreated with amyloid-β_1-40 _peptide) (n = 6 independent experiments). **P < 0.01 as compared with control, unpaired *t*-test. (**B**) Cells were treated with amyloid-β_1-40 _peptide (5 μM) in the absence (Control) and presence of DLPhtEtn at concentrations as indicated in serum-free extracellular solution for 48 h, and cell viability was assayed. Data represents the mean (± SEM) percentage of basal levels (MTT intensities of cells untreated with amyloid-β_1-40 _peptide) (n = 6 independent experiments). *P < 0.1, **P < 0.01 as compared with control, unpaired *t*-test. (**C**) Cells were treated with thapsigargin (100 nM) in the absence (Control) and presence of DLPhtEtn (30 μM) in serum-free extracellular solution for 24 h, and cell viability was assayed. Data represents the mean (± SEM) percentage of basal levels (MTT intensities of cells untreated with amyloid-β_1-40 _peptide) (n = 6 independent experiments). *P *value, unpaired *t*-test.

Amyloid-β peptide, a neurotoxic factor for Alzheimer disease, mediates ER stress-induced apoptosis [42]. Amyloid-β_1-40 _peptide reduced PC12 cell viability in a concentration (0.2-20 μM)-dependent manner at 48-h treatment and the maximal effect was obtained with 5 μM (data not shown). We, therefore, examined the effect of a variety of phospholipids on PC-12 cell death induced by amyloid-β_1-40 _peptide at 5 μM. Treatment with amyloid-β_1-40 _peptide (5 μM) for 48 h reduced PC-12 cell viability to approximately 40% of basal levels (Figure 1A). Of bath-applied phospholipids used here such as DLPhtEtn, LPPhtEtn, DOPhtEtn, DPPhtEtn, DHPhtEtn, DSPhtEtn, and DLPhtCho at a concentration of 30 μM, DLPhtEtn alone significantly prevented amyloid-β_1-40 _peptide-induced PC-12 cell death (Figure 1A). The DLPhtEtn effect was concentration (1-300 μM)-dependent, reaching the maximum at 30 μM (Figure 1B).

Thapsigargin, depleting Ca^2+ ^from the ER, is shown to cause ER stress-induced apoptosis in PC-12 cells [43]. We subsequently examined the effect of DLPhtEtn on PC-12 cell death induced by thapsigargin. Treatment with thapsigargin (100 nM) for 24 h reduced cell viability to nearly 20% of basal levels, and bath-application with DLPhtEtn (30 μM) significantly inhibited thapsigargin-induced PC-12 cell death (Figure 1C). Collectively, these results indicate that DLPhtEtn protects PC-12 cells from apoptotic cell death induced by amyloid-β_1-40 _peptide or thapsigargin.

### DLPhtEtn reverses hyper-motor activities for SAMP8 mice

**Figure 2:**
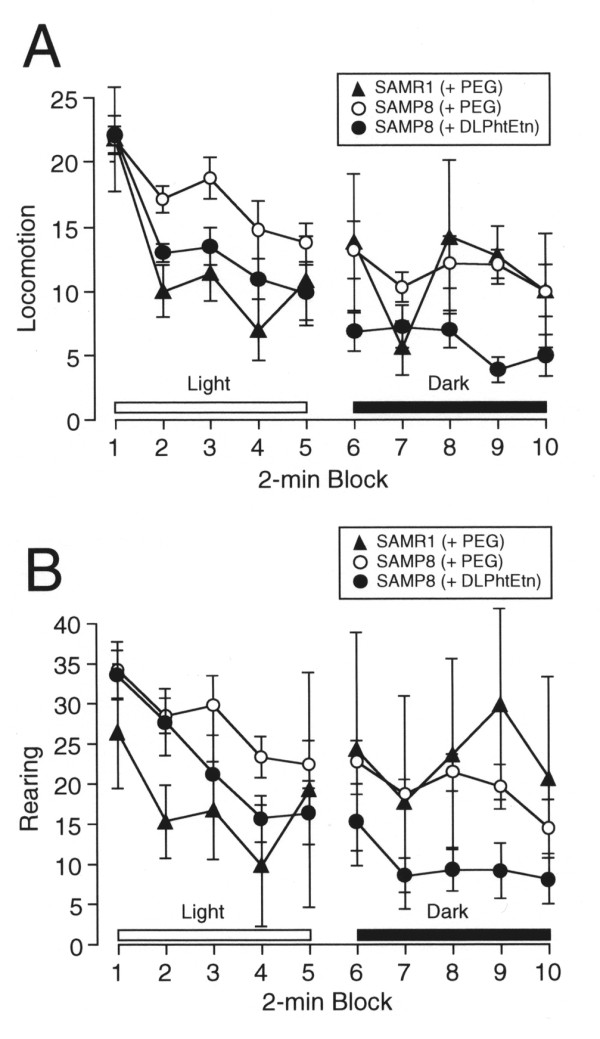
**The effect of DLPhtEtn on motor activity**. Open field test was carried out in SAMR1 and SAMP8 mice after oral administration with PEG or DLPhtEtn (1 mg/kg) for 7 months. The results of locomotion and rearing activities are shown in (**A**) and (**B**), respectively. Values represent the mean (± SEM) number of times from 2-min block (n = 5 for SAMR1 mice treated with PEG, 10 for SAMP8 mice treated with PEG, and 7 for SAMP8 mice treated with DLPhtEtn).

We next carried out a series of behaviors for mice treated with PEG (per os) or DLPhtEtn (per os) for 7 months, that include motor activities, spatial learning and memory, and fear memory. In the open field test to assess motor activities, locomotion activity for SAMP8 mice treated with PEG increased as compared with the activity for SAMR1 mice treated with PEG, dominantly under the light condition (*P *= 0.0004, *F *= 8.526 among SAMR1+PEG group, SAMP8+PEG group, and SAMP8+DLPhtEtn group for ANOVA test; *P *= 0.0007 between SAMR1+PEG group and SAMP8+PEG group for Fisher's PLSD test)(Figure 2A). Rearing activity for SAMP8 mice treated with PEG also increased as compared with the activity for SAMR1 mice treated with PEG, dominantly under the light condition (*P *< 0.0001, *F *= 10.638 among SAMR1+PEG group, SAMP8+PEG group, and SAMP8+DLPhtEtn group for ANOVA test; *P *< 0.0001 between SAMR1+PEG group and SAMP8+PEG group for Fisher's PLSD test)(Figure 2B). These results indicate that SAMP8 mice exhibit more excess motor activities than SAMR1 mice.

Locomotion activity for SAMP8 mice treated with DLPhtEtn (1 mg/kg) was significantly depressed as compared with the activity for SAMP8 mice untreated with DLPhtEtn under both the light and dark conditions (*P *= 0.0011 under the light condition and *P *< 0.0001 under the dark condition between SAMP8+PEG group and SAMP8+DLPhtEtn group for Fisher's PLSD test)(Figure 2A). Rearing activity for SAMP8 mice treated with DLPhtEtn (1 mg/kg) was also significantly suppressed as compared with the activity for SAMP8 mice untreated with DLPhtEtn under both the light and dark conditions (*P *= 0.0010 under the light condition and *P *< 0.0001 under the dark condition between SAMP8+PEG group and SAMP8+DLPhtEtn group for Fisher's PLSD test)(Figure 2B). DLPhtEtn, thus, reverses hyper-motor activities for SAMP8 mice.

### DLPhtEtn improves spatial memory impairment for SAMP8 mice

**Figure 3:**
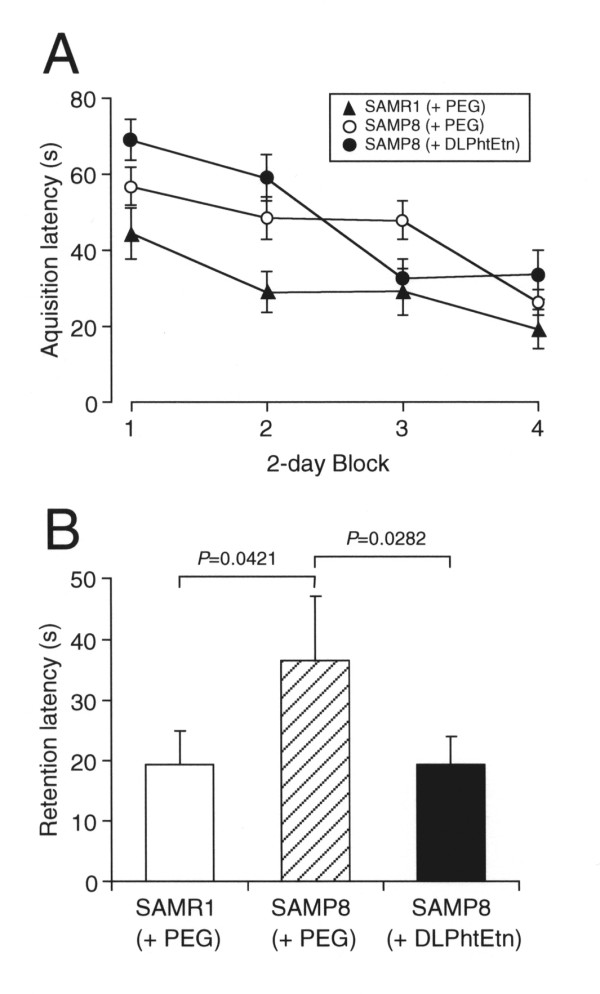
**The effect of DLPhtEtn on spatial learning and memory**. (**A**) Water maze test was carried out in SAMR1 and SAMP8 mice after oral administration with PEG or DLPhtEtn (1 mg/kg) for 7 months, and the acquisition latency was measured. Values represent the mean (± SEM) acquisition latency from 2-day block (n = 5 for SAMR1 mice treated with PEG, 10 for SAMP8 mice treated with PEG, and 7 for SAMP8 mice treated with DLPhtEtn). (**B**) Seven days later, the retention latency was measured. Values represent the mean (± SEM) retention latency from 2-day block (n = 5 for SAMR1 mice treated with PEG, 10 for SAMP8 mice treated with PEG, and 7 for SAMP8 mice treated with DLPhtEtn). *P *values, unpaired *t*-test.

In the water maze test to assess spatial learning and memory, the acquisition latency for SAMP8 mice treated with PEG was significantly delayed as compared with the latency for SAMR1 mice treated with PEG (*P *= 0.0003, *F *= 8.343 among SAMR1+PEG group, SAMP8+PEG group, and SAMP8+DLPhtEtn group for ANOVA test; *P *= 0.0009 between SAMR1+PEG group and SAMP8+PEG group for Fisher's PLSD test)(Figure 3A). DLPhtEtn (1 mg/kg) had no significant effect on the prolonged latency for SAMP8 mice untreated with DLPhtEtn (Figure 3A), suggesting no beneficial action of DLPhtEtn on age-related spatial learning disorders.

The retention latency for SAMP8 mice treated with PEG was significantly longer than the latency for SAMR1 mice treated with PEG (*P *= 0.0482, *F *= 3.687 among SAMR1+PEG group, SAMP8+PEG group, and SAMP8+DLPhtEtn group for ANOVA test; *P *= 0.0421 between SAMR1+PEG group and SAMP8+PEG group for Fisher's PLSD test), and DLPhtEtn (1 mg/kg) significantly shortened the prolonged latency for SAMP8 mice untreated with DLPhtEtn, reaching levels similar to the latency for SAMR1 mice treated with PEG (Figure 3B). This implies that DLPhtEtn could ameliorate age-related spatial memory impairment.

### DLPhtEtn does not affect fear memory impairment for SAMP8 mice

**Figure 4:**
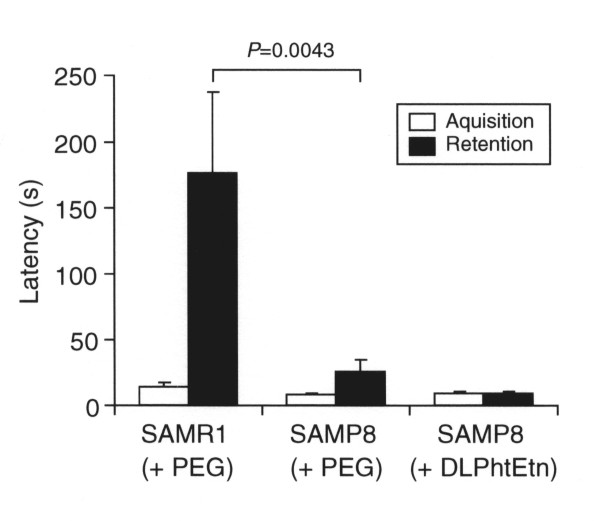
**The effect of DLPhtEtn on fear memory**. In the passive avoidance test, the acquisition latency was measured in SAMR1 and SAMP8 mice after oral administration with PEG or DLPhtEtn (1 mg/kg) for 7 months, and 24 h later the retention latency was monitored. Values represent the mean (± SEM) latency (n = 5 for SAMR1 mice treated with PEG, 10 for SAMP8 mice treated with PEG, and 7 for SAMP8 mice treated with DLPhtEtn). *P *value, unpaired *t*-test.

In the passive avoidance test to assess fear memory, there was no significant difference in the acquisition latency between SAMR1 and SAMP8 mice treated with PEG, and DLPhtEtn (1 mg/kg) had no effect on the latency for SAMP8 mice (Figure 4).

The retention latency for SAMR1 mice treated with PEG was significantly longer than the latency for SAMP8 mice treated with PEG (Figure 4), indicating fear memory impairment for SAMP8 mice. DLPhtEtn (1 mg/kg) shortened the retention latency for SAMP8 mice, but not significantly (Figure 4). This suggests that DLPhtEtn does not influence fear memory impairment for SAMP8 mice.

### DLPhtEtn protects hippocampal neuronal death for SAMP8 mice

**Figure 5:**
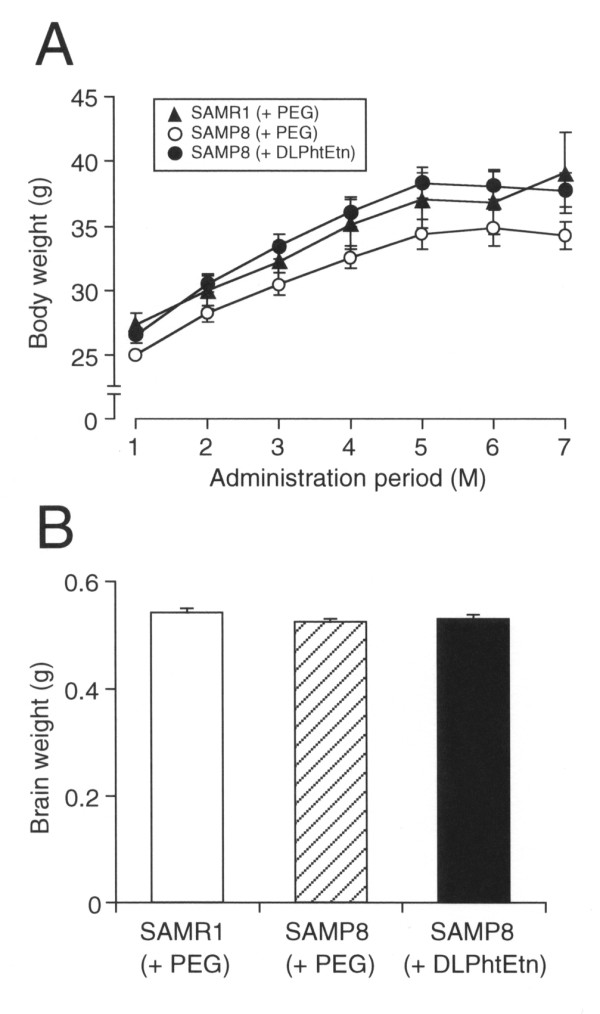
**Effects of DLPhtEtn on the body and brain weight**. (**A**) The body weight for SAMR1 and SAMP8 mice was measured every one month from the beginning of oral administration with PEG or DLPhtEtn (1 mg/kg) through 7 months. Values represent the mean (± SEM) body weight (n = 5 for SAMR1 mice treated with PEG, 10 for SAMP8 mice treated with PEG, and 7 for SAMP8 mice treated with DLPhtEtn). (**B**) The weight of the brain, isolated from SAMR1 and SAMP8 mice after a series of behavioral tests, was measured. Values represent the mean (± SEM) brain weight (n = 5 for SAMR1 mice treated with PEG, 10 for SAMP8 mice treated with PEG, and 7 for SAMP8 mice treated with DLPhtEtn).

Our final attempt was to see whether DLPhtEtn exerts its protective action against age-related neuronal death for SAMP8 mice. A gradual increase in the body weight both for SAMP8 and SAMR1 mice was found with oral administration with PEG or DLPhtEtn (1 mg/kg) throughout 7 months (Figure 5A). The increase for SAMP8 mice treated with DLPhtEtn was significantly greater than the increase for SAMP8 mice untreated with DLPhtEtn (*P *= 0.0012, *F *= 7.046 among SAMR1+PEG group, SAMP8+PEG group, and SAMP8+DLPhtEtn group for ANOVA test; *P *= 0.0143 between SAMP8+PEG group and SAMP8+DLPhtEtn group for Fisher's PLSD test), but there was no significant difference in the increase between for SAMP8 mice treated with DLPhtEtn and SAMR1 mice treated with PEG (Figure 5A).

**Figure 6:**
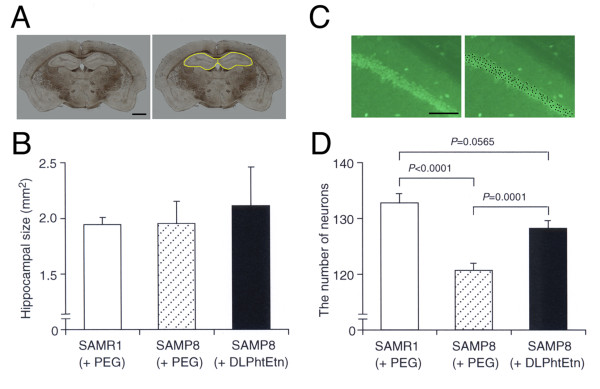
**Effects of DLPhtEtn on hippocampal size and the number of hippocampal neurons**. (**A**) Eight coronal sections were prepared from the brain for SAMR1 and SAMP8 mice after a series of behavioral tests. Hippocampal area (yellow lines in the right column) both on the left and right sides was measured, and areas from 8 sections per a mouse were summated. Bar, 1 mm. (**B**) Values represent the mean (± SEM) hippocampal size (n = 5 for SAMR1 mice treated with PEG, 10 for SAMP8 mice treated with PEG and 7 for SAMP8 mice treated with DLPhtEtn). (**C**) For coronal sections from the brain for SAMR1 and SAMP8 mice with oral administration with PEG or DLPhtEtn (1 mg/kg) for 7 months, the number of neurons immunoreactive to an anti-NeuN antibody was counted in the consistent area (142 μm × 192 μm) of the hippocampal CA1 region both on the left and right sides (black dots in the right column) from 8 sections per a mouse and summated. Bar, 50 μM. (**D**) Values represent the mean (± SEM) number of hippocampal neurons (n = 5 for SAMR1 mice treated with PEG, 10 for SAMP8 mice treated with PEG and 7 for SAMP8 mice treated with DLPhtEtn). *P *values, unpaired *t*-test.

No significant difference in the brain weight was found between SAMR1 and SAMP8 mice treated with PEG, and DLPhtEtn (1 mg/kg) had no effect on the brain weight for SAMP8 mice (Figure 5B). Likewise, there was no significant difference in the hippocampal size between SAMR1 and SAMP8 mice treated with PEG, and DLPhtEtn (1 mg/kg) did not affect the hippocampal size for SAMP8 mice (Figure 6A,6B). Amazingly, the number of hippocampal neurons for SAMP8 mice treated with PEG significantly decreased as compared with that for SAMR1 mice treated with PEG (Figure 6C,6D). DLPhtEtn (1 mg/kg) significantly prevented a decrease in the number of hippocampal neurons for SAMP8 mice untreated with DLPhtEtn (Figure 6C,6D). This accounts for the protective effect of DLPhtEtn against age-related hippocampal neuronal death.

## Discussion

In the *in vitro *systems, bath-application with DLPhtEtn inhibited PC-12 cell death induced by amyloid-β_1-40 _peptide in a concentration (1-300 μM)-dependent manner, but such effect was not obtained with the other phospholipids, LPPhtEtn, DOPhtEtn, DPPhtEtn, DHPhtEtn, DSPhtEtn, and DLPhtCho. Bath-application with DLPhtEtn also prevented PC-12 cell death induced by thapsigargin. It is suggested from these results that DLPhtEtn prevents ER stress-induced neuronal apoptotic cell death. In the *in vivo *systems, oral administration with DLPhtEtn inhibited a decrease in the number of hippocampal neurons for SAMP8 MICE, although the lipid had no effect on the brain weight and the hippocampal size. This indicates that DLPhtEtn still exerts its protective action against neuronal death in the *in vivo *systems. Then, we thought that DLPhtEtn could improve age-related cognitive decline.

In the water maze test, the acquisition and retention latencies for SAMP8 mice treated with PEG were significantly longer than the latencies for SAMR1 mice treated with PEG. In the open field test, SAMP8 mice exhibited more excess motor activities than SAMR1 mice. This indicates that the prolonged acquisition and prolonged latencies for SAMP8 mice untreated with DLPhtEtn in the water maze test is not due to reduced motor activities, i.e., reduced swimming speed, even though the swimming speed was not actually measured here. Accordingly, this confirms age-related decline of spatial learning and memory for SAMP8 mice. In the water maze test, oral administration with DLPhtEtn significantly shortened the prolonged retention latency for SAMP8 mice untreated with DLPhtEtn, reaching levels similar to the latency for SAMR1 mice, although DLPhtEtn had no efficient effect on the prolonged acquisition latency. This, taken together with the result that DLPhtEtn depressed hyper-motor activity for SAMP8 mice in the open field test, indicates that the shortened retention latency for SAMP8 mice treated with DLPhtEtn in the water maze test is not due to enhanced motor activities, i.e., accelerated swimming speed. DLPhtEtn, thus, appears to ameliorate age-related decline of spatial memory.

A study has shown that impairment of spatial learning and memory disorders for SAMP mice is due to altered fear memory rather than cognitive impairment [44]. In the passive avoidance test, the retention latency for SAMP8 mice treated with PEG was significantly shorter than that for SAMR1 mice treated with PEG, while there was no significant difference in the acquisition latency between SAMP8 and SAMR1 mice. Oral administration with DLPhtEtn had no effect on both the acquisition and retention latencies for SAMP8 mice, excluding the DLPhtEtn action on fear memory. This implies that DLPhtEtn improves spatial memory deterioration for SAMP8 mice, regardless of fear memory alteration.

Overall, the results of the present study show that DLPhtEtn ameliorates age-related spatial memory decline, possibly by protecting hippocampal neuronal death. This suggests that DLPhtEtn could be developed as a promising drug against senile dementia. ER stress-induced neuronal apoptosis is the major factor for pathogenesis of neurodegenerative diseases such as Alzheimer disease and Parkinson disease [4-7,9]. DLPhtEtn rescued PC-12 cells from apoptosis induced by amyloid-β_1-40 _peptide or thapsigargin. This, in the light of the fact that amyloid-β peptide or thapsigargin causes ER stress-induced apoptosis [42,43], raises the possibility that DLPhtEtn could also exert its beneficial action against dementia associated with neurodegenerative diseases.

In conclusion, the results of the present study show that DLPhtEtn is capable of improving age-related spatial memory deterioration, at least in part by preventing neuronal cell death.

## Abbreviations

ER: Endoplasmic reticulum; DLPhtEtn: 1,2-dilinoleoyl-*sn*-glycero-3-phosphoethanolamine; PEG: polyethylene glycol; SAMP8: senescence-accelerated mouse-prone 8; S1P: sphingosine 1-phosphate; DLPhtCho: 1,2-dilinoleoyl-*sn*-glycero-3-phosphocholine; LTP: long-term potentiation; ACh: acetylcholine; PKC: protein kinase C; DCP-LA: 8-[2-(2-pentyl-cyclopropylmethyl)-cyclopropyl]-octanoic acid; SAMR: senescence-accelerated mouse-resistant; LPPhtEtn: 1-linoleoyl-2-palmitoyl-*sn*-glycero-3-phosphoethanolamine; DOPhtEtn: 1,2-dioleoyl-*sn*-glycero-3-phosphoethanolamine; DPPhtEtn: 1,2-dipalmitoyl-*sn*-glycero-3-phosphoethanolamine; DHPhtEtn: 1,2-diheptadecanoyl-*sn*-glycero-3-phosphoethanolamine; DSPhtEtn: 1,2-distearoyl -*sn*-glycero-3-phosphoethanolamine; MTT: 3-(4,5-dimethyl-2-thiazolyl)-2,5-diphenyl-2H-tetrazolium bromide; ANOVA: analysis of variance; PLSD: Protected Least Significant Difference

## Competing interests

The authors declare that they have no competing interests.

## Authors' contributions

All authors read and approved the final manuscript.

TY carried out all the experiments except for a series of behavioral tests.

TN carried out a series of behavioral tests.

TN is responsible for conception and design, analysis and interpretation of the experimental data, drafting, revising and approval of the manuscript.
